# Effect of Improved Water Quality, Sanitation, Hygiene and Nutrition Interventions on Respiratory Illness in Young Children in Rural Bangladesh: A Multi-Arm Cluster-Randomized Controlled Trial

**DOI:** 10.4269/ajtmh.19-0769

**Published:** 2020-02-24

**Authors:** Sania Ashraf, Mahfuza Islam, Leanne Unicomb, Mahbubur Rahman, Peter J. Winch, Benjamin F. Arnold, Jade Benjamin-Chung, Pavani K. Ram, John M. Colford, Stephen P. Luby

**Affiliations:** 1 International Centre for Diarrhoeal Disease Research, Bangladesh (icddr,b), Dhaka, Bangladesh;; 2 Department of International Health, Johns Hopkins Bloomberg School of Public Health, Baltimore, Maryland;; 3 Division of Epidemiology and Biostatistics, School of Public Health, University of California Berkeley, Berkeley, California;; 4 School of Public Health and Health Professions, University at Buffalo, Buffalo, New York;; 5 Division of Infectious Diseases and Geographic Medicine, Stanford University, Stanford, California

## Abstract

Acute respiratory infections cause mortality in young children. We assessed the effects of water, sanitation, hygiene (WASH) and nutritional interventions on childhood ARI. Geographic clusters of pregnant women from rural Bangladesh were randomly assigned to receive 1) chlorinated drinking water and safe storage (W); 2) upgraded sanitation (S); 3) handwashing promotion (H); 4) combined water, sanitation, and handwashing (WSH); 5) nutrition intervention including lipid-based nutrient supplements; 6) combined WSH plus nutrition (WSHN); or 7) no intervention (control). Masking of participants was not possible. Acute respiratory illness was defined as caregiver-reported persistent cough, panting, wheezing, or difficulty breathing in the past 7 days among index children, those born to enrolled women. We assessed outcomes at 12 and 24 months of intervention using intention to treat. Compared with children in the control group (ARI prevalence, *P*: 8.9%), caregivers of index children reported significantly lower ARI in the water (*P*: 6.3%, prevalence ratio (PR): 0.71; 95% CI: 0.53, 0.96), sanitation (*P*: 6.4%, PR: 0.75, 95% CI: 0.58, 0.96), handwashing (*P*: 6.4%, PR: 0.68, 95% CI: 0.50, 0.93), and the combined WSH+N arms (*P*: 5.9%, PR: 0.67, 95% CI: 0.50, 0.90). Those in the nutrition (*P*: 7.4%, PR: 0.84, 95% CI: 0.63, 1.10) or the WSH arm (*P*: 8.9%, PR: 0.99, 95% CI: 0.76, 1.28) reported similar ARI prevalence compared with control children. Single targeted water, sanitation, and hygiene interventions reduced reported respiratory illness in young children. There was no apparent respiratory health benefit from combining WASH interventions.

## INTRODUCTION

Acute respiratory infections (ARIs) are a leading cause of morbidity and mortality in young children globally.^1^ Acute respiratory infection and pneumonia cause the majority of hospitalizations and death among children younger than 5 years especially in low-income countries.^2^ Risk factors for pneumonia include low birth weight, malnutrition, low exclusive breastfeeding rates, poor handwashing, crowding, use of solid fuels, and low maternal education, all of which are common in poor households.^2^ In low-income settings, effective interventions include immunization against respiratory pathogens (measles, *Haemophilus influenzae* type B, and pneumococcus) and reducing indoor air pollution.^3^ However, poor environmental conditions that support transmission of respiratory pathogens can worsen childhood morbidity.^4^ Water, sanitation, hygiene (WASH) interventions that improve these conditions, therefore, have the potential to reduce respiratory illness by interrupting pathogen transmission.

Handwashing with water and/or soap effectively interrupts transmission of respiratory pathogens through droplets and fomites.^5^ Older observational studies noted reductions in child mortality from pneumonia after the introduction of improved water quality through centralized drinking water interventions.^6^ Nutrition interventions such as promoting exclusive breastfeeding or delivering vitamin A that boost a child’s immunity can alleviate childhood morbidities including respiratory illness.^7^ Water, sanitation, hygiene and nutrition interventions that reduce diarrheal disease morbidity can also reduce pneumonia by preventing compromised immune responses or micronutrient deficiencies, especially in already malnourished children.^8^ Combined school-based interventions that improved water quality and sanitation were associated with reductions in respiratory illness.^9,10^ These overlapping risk factors suggest that combining interventions that improve nutrition and with those that improve water quality, sanitation, and hygiene conditions in resource-poor settings could lead to larger reductions in childhood illness compared with each component alone.^11^

Although the impact of improved WASH and nutrition on childhood respiratory health has been studied, their impact has not been directly compared with each other individual intervention or an intervention that combined WASH and nutrition in the same study population. Because combined interventions are often more difficult and expensive to implement, compared with single interventions, determining the relative health effects of each can help identify cost-effective strategies. We aimed to assess whether the effect of single WASH and nutrition interventions reduced respiratory illness in young children when delivered alone or in combination in the WASH Benefits trial in rural Bangladesh.

## METHODS

### Study design.

The WASH Benefits Bangladesh study was a community-based cluster-randomized trial conducted in rural villages in Gazipur, Kishoreganj, Mymensingh and Tangail districts. The study design and rationale were published earlier (See Consolidated Standards of Reporting Trials checklist in supporting documents).^12^

It included six intervention arms and a double-sized control arm. In Bangladesh, the unit of randomization was a group of compounds visited by a single local promoter and separated by at least a 1-km buffer region to minimize the risk of spillover between clusters. The clusters were block randomized into either one of the six intervention arms or the control arm.^13^

The study protocol was approved by the Research and Ethical Review Committee at the International Centre for Diarrhoeal Disease Research, Bangladesh (PR-11063), the University of California, Berkeley (2011-09-3652), and the Institutional Review Board at Stanford University (25863).

### Participants.

Research assistants screened rural compounds to identify eligible pregnant women in their first or second trimester who did not plan to move in the next 24 months. Pregnant women who lived close to each other were enrolled into the study following written informed consent from the compound head, the woman, and guardians of children younger than 3 years. The children born to the enrolled pregnant women were considered “index” children. We followed the closed cohort longitudinally and measured symptoms of illness at 12 and 24 months after initiating the intervention.

### Randomization and masking.

Blocks of eight adjacent clusters were randomized into 1) chlorinated drinking water and safe water storage, 2) sanitation, 3) handwashing, 4) combined water + sanitation + handwashing (WSH), 5) nutrition, 6) combined nutrition + WSH, or the 7) nonintervention control group. The control arm was double sized to improve precision of estimates when compared with multiple arms. An offsite investigator (B. F. A.) used a random number generator to block randomize these clusters. This trial was designed as a pair-matched, cluster-randomized trial. This was a geographically pair-matched design meaning any comparison between two arms is pair-matched within the randomization block. The participants were unaware of their intervention group assignment until after the baseline survey and randomization. Because the intervention included distribution of products and related promotion by community health promoters, masking of the subjects or the data collectors was not possible. The research team who implemented the intervention was separate from the data collection team. The analysis was carried out using re-randomized uninformative assignments to enable masked statistical analyses from raw datasets. Results were unmasked once statistical analysis was completed.

### Procedures.

The interventions were described in detail previously.^12,13^ Interventions were delivered at the household level or the compound level and included 1) chlorine tablets and safe storage vessel; 2) upgrades to dual-pit latrines with water seals for all households in the compound and provision of child potties and sani-scoops to index households; 3) handwashing stations with soapy water detergent and bottles near the kitchen and the latrine delivered to index households; 4) age-appropriate nutrition from birth to 24 months including a supply of lipid-based nutrient supplements (6–24 months) in addition to exclusive breastfeeding and maternal and infant nutrition recommendations to mothers and the index child; 5) combined WSH; and 6) combined WSH plus nutrition (WSH+N). Local women from the community were recruited and trained as promoters who conducted household visits and community discussions to promote the interventions based on a behavior-change strategy. These promotions included interactive sessions to develop collaborative solutions with the participants to continue their improved practices. The promoters were paid a monthly stipend of approximately USD 20. Control arms did not receive any hardware/products or promoter visits.

### Outcomes.

In this study, we assessed the impact on respiratory outcomes in index children as reported by the primary caregiver. We asked the primary caregiver to recall if the index child had the following symptoms: 1) persistent cough, 2) panting/wheezing/difficulty breathing, or 3) fever during specific days in the last week. The study team did not collect data on clinical signs of the severity of the respiratory syndrome. In this study, we used combinations of these three reported symptoms to assess childhood respiratory illness.

Our main outcome of interest was a 7-day prevalence of acute respiratory illness (ARI), defined as caregiver-reported symptoms of persistent cough or panting, wheezing, or difficulty breathing (1 or 2) in the 7 days before the interview.^14^ In secondary analyses, we explored the impact of these interventions using alternate combinations of the measured symptoms: 7-day prevalence of only panting, wheezing, or difficulty breathing (2) and ARI plus fever ([1 or 2] and 3). Panting, wheezing, or difficulty breathing encompass symptoms of asthma, bronchiolitis, or, occasionally, bacterial pneumonia. Although this might reflect chronic conditions such as asthma, these symptoms reflect a burden and a risk factor for respiratory illness morbidity. Exploring ARI plus fever could be indicative of more severe respiratory infection. Respiratory illness and these definitions were not prespecified for this trial.

Trained field surveyors who were not involved in the delivery of the interventions interviewed the mother of the index child to collect data on respiratory symptoms. We included caregiver-reported abrasion or bruising as negative control outcomes.^15^ Outcomes were measured approximately 12 and 24 months following intervention roll out. These outcome evaluations were spread out over the entire year because of the long duration of enrollment. Intervention adherence was assessed by a separate team at regular intervals using structured observations and objective measures.^16^

### Statistical analyses.

The sample size calculation for this trial was based on primary outcomes, diarrhea, and child growth. It assumed a relative risk of diarrhea of 0.7 or smaller, with 10% prevalence in the control group, and a difference of 0.15 length for age Z-score between the intervention and control groups, adjusting for repeated measures within clusters. Other assumptions were type I error (α) of 0.05, power (1−β) of 0.8, and a 10% dropout after baseline. The control arm was double sized to account for multiple hypothesis tests.^12^

We conducted an intention to treat analysis in which we compared each intervention arm against the control arm. Because the nutrition intervention provided supplements only to the index child, we restricted the analysis to index children for all arms. We conducted two subgroup analyses 1) stratified by child gender because male children might be more susceptible to respiratory illness^3^ and 2) by survey round (Year 1 and Year 2) to examine differences in intervention effects on the prevalence of reported respiratory illness overtime. We also compared the impact on outcomes between combined WSH and individual arms and the nutrition plus WSH (WSHN) arm and WSH and the nutrition-only arm.

The analysis followed the procedures used in the primary outcome analysis (pre-registered analysis protocol https://osf.io/wvyn4/). The pair-matched design ensured that the calendar time of the measurements (season) was balanced across treatment groups.

We used a generalized log linear regression model to estimate the effect of each intervention compared with the control group. To estimate adjusted prevalence ratios (PRs), we included prespecified covariates that were associated with the outcome based on a likelihood ratio test (*P* < 0.2). Potential covariates included field staff who collected data, including the month of measurement, household food insecurity, child age, child gender, mother’s age, mother’s height, mother’s education level, number of children < 18 years in the household, number of individuals living in the compound, distance in minutes to the primary water source, household roof, floor, wall materials, and household assets. Analyses were carried out with R, version 3.2.4 (R Foundation for Statistical Computing, Vienna, Austria) and STATA 13.0 (Stata Corp LP, College Station, TX).

The trial is registered with ClinicalTrials.gov, NCT01590095. Independent data safety monitoring boards in Bangladesh oversaw the trial.

## RESULTS

**Figure 1.**
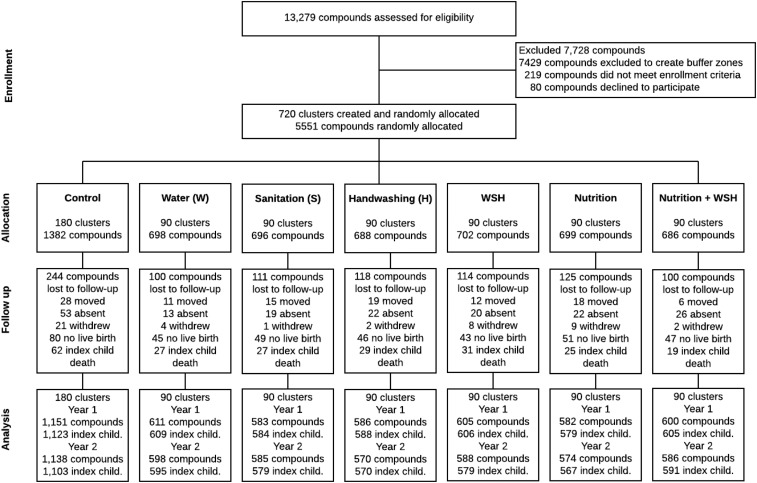
Summary of participant enrollment, randomization, retention, and analysis populations for respiratory outcomes, that is, index children.

Field-workers recruited participants from 5,551 compounds to form 720 clusters of pregnant women. Between May 2012 and July 2013, we randomly allocated clusters to one of six interventions or the double-sized control arm (Figure 1). Loss to follow-up included no live births (*n* = 361), death of index child (*n* = 235), relocation (*n* = 375), withdrawal (*n* = 296), and absence during assessments (*n* = 182) (Figure 1). Treatment groups were balanced at baseline on demographic characteristics, household composition, facilities and practices relating to the use of cooking fuel, drinking water, handwashing, and sanitation (Table 1). Specifically, the average number of household members was five. A majority used a shallow tube well for their drinking water (74%). On average, less than a third of the households owned hygienic latrines with functional water seals (29%). Availability of water or soap was low near the toilet or the kitchen. Overall, 69% of households reported that they did not face food insecurity. Symptoms for respiratory illness was assessed for 4,747 index children at the 12-month follow-up (mean age: 0.73 years, SD: 0.14) and 4,667 index children (mean age: 1.87 years, SD: 0.17) at the 24-month follow-up.

**Table 1:** Baseline characteristics across intervention arms

	Control	Water	Sanitation	Handwashing	Water + sanitation + handwashing	Nutrition	Nutrition + water + sanitation + handwashing
No. of households	*N* = 1,382	*N* = 698	*N* = 696	*N* = 688	*N* = 702	*N* = 699	*N* = 686
Maternal							
Age (years)	23.6 (5.0)	23.7 (5.2)	23.7 (5.2)	23.8 (5.5)	24.3 (5.5)	23.7 (5.1)	23.8 (5.5)
Years of education	5.9 (3.4)	5.8 (3.4)	5.8 (3.5)	5.8 (3.3)	5.9 (3.3)	5.8 (3.5)	5.6 (3.5)
Paternal							
Years of education	4.9 (4.0)	4.9 (4.1)	5.0 (4.2)	4.6 (4.1)	5.0 (4.2)	4.8 (4.0)	4.7 (3.9)
Works in agriculture	414 (30%)	224 (32%)	204 (29%)	249 (36%)	216 (31%)	232 (33%)	207 (30%)
Household							
Number of people	4.7 (2.3)	4.6 (2.2)	4.7 (2.1)	4.7 (2.2)	4.7 (2.1)	4.7 (2.2)	4.7 (2.1)
Has electricity	784 (57%)	422 (60%)	408 (59%)	405 (59%)	426 (61%)	409 (59%)	412 (60%)
Has a cement floor	145 (10%)	82 (12%)	85 (12%)	55 (8%)	77 (11%)	67 (10%)	72 (10%)
Acres of agricultural land owned	0.15 (0.21)	0.14 (0.20)	0.14 (0.22)	0.14 (0.20)	0.15 (0.23)	0.16 (0.27)	0.14 (0.38)
Drinking water							
Tube well as primary water source	1,038 (75%)	500 (72%)	519 (75%)	482 (70%)	546 (78%)	519 (74%)	504 (73%)
Stored water observed at home	666 (48%)	353 (51%)	341 (49%)	347 (50%)	304 (43%)	301 (43%)	331 (48%)
Sanitation							
Daily defecation in the open							
Adult men	97 (7%)	39 (6%)	52 (8%)	64 (9%)	54 (8%)	59 (9%)	50 (7%)
Adult women	62 (4%)	18 (3%)	33 (5%)	31 (5%)	29 (4%)	39 (6%)	24 (4%)
Children aged 8 to < 15 years	53 (10%)	25 (9%)	28 (9%)	43 (15%)	30 (10%)	23 (8%)	28 (10%)
Children aged 3 to < 8 years	267 (38%)	141 (37%)	137 (38%)	137 (39%)	137 (38%)	129 (39%)	134 (37%)
Children aged 0 to < 3 years	245 (82%)	112 (85%)	117 (84%)	120 (85%)	123 (79%)	128 (85%)	123 (88%)
Latrine							
Owned	750 (54%)	363 (52%)	374 (54%)	372 (54%)	373 (53%)	377 (54%)	367 (53%)
Concrete slab	1,251 (95%)	644 (95%)	610 (92%)	613 (93%)	620 (93%)	620 (94%)	621 (94%)
Functional water seal	358 (31%)	183 (31%)	177 (30%)	162 (28%)	152 (26%)	183 (31%)	155 (27%)
Visible stool on slab or floor	625 (48%)	350 (53%)	332 (52%)	335 (52%)	289 (44%)	331 (51%)	298 (46%)
Owned a potty	61 (4%)	27 (4%)	28 (4%)	35 (5%)	27 (4%)	36 (5%)	30 (4%)
Human feces observed							
In the house	114 (8%)	65 (9%)	56 (8%)	70 (10%)	48 (7%)	58 (8%)	49 (7%)
In the child’s play area	21 (2%)	6 (1%)	6 (1%)	8 (1%)	7 (1%)	8 (1%)	7 (1%)
Handwashing							
Within six steps of latrine							
Has water	178 (14%)	83 (13%)	81 (13%)	63 (10%)	67 (10%)	62 (10%)	72 (11%)
Has soap	88 (7%)	50 (8%)	48 (8%)	34 (5%)	42 (7%)	32 (5%)	36 (6%)
Within six steps of kitchen							
Has water	118 (9%)	51 (8%)	51 (8%)	45 (7%)	61 (9%)	61 (9%)	60 (9%)
Has soap	33 (3%)	18 (3%)	14 (2%)	13 (2%)	15 (2%)	23 (3%)	18 (3%)
Nutrition							
Household is food secure*	932 (67%)	495 (71%)	475 (68%)	475 (69%)	482 (69%)	479 (69%)	485 (71%)

Data are expressed in *n* (%) or mean (SD). Percentages were estimated from slightly smaller denominators than those shown at the top of the table for the following variables because of missing values: father works in agriculture, open defecation, latrine has a concrete slab, latrine has a functional water seal, visible stool on the latrine slab or floor, ownership of child potty, observed feces in the house or child’s play area, and handwashing variables.

* Assessed by the Household Food Insecurity Access Scale.

This trial achieved high adherence to all interventions.^13,16^ All measures suggested marked differences in promoted behaviors from the control group at both Year 1 and Year 2, with adherence over 75% in the single intervention group and the combined intervention groups.

**Figure 2.**
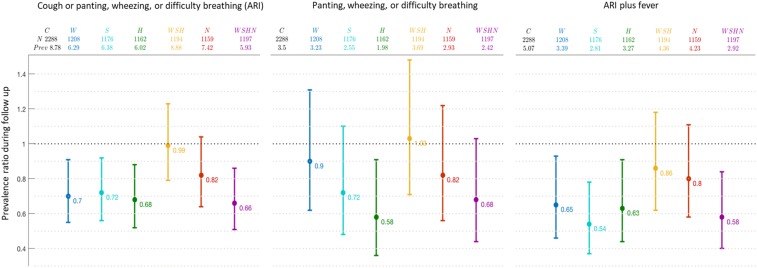
Intervention effects on the 7-day prevalence of respiratory illness in index children; 1- and 2-year assessments combined in Bangladesh. Acute respiratory illness (ARI) defined as mothers’ reports of persistent cough or panting, wheezing, or difficulty breathing in the past 7 days among index children. Data are prevalence ratios compared with the children in the control group, with 95% CIs. C = control; H = handwashing; S = sanitation; W = water; WSH = combined water, sanitation, and handwashing; WSHN = water, sanitation, handwashing, and nutrition.

Compared with the control group (8.9%), the reported prevalence of ARI in index children was lower in the water (6.3%; PR: 0.71, 95% CI: 0.55, 0.91), sanitation (6.4%; PR: 0.72, 95% CI: 0.56, 0.92), handwashing (6.4%; PR: 0.68, 95% CI: 0.52, 0.88), and the combined WSH+N arms (5.9%; PR: 0.66, 95% CI: 0.51, 0.86) at 1-year and 2-year follow-ups (Figure 2). Notably, the impact observed in WSH+N was similar to that in the single WSH arms. In our study, the reported ARI prevalence in index children from the nutrition (7.4%; PR: 0.82, 95% CI: 0.64, 1.04) or the combined WSH arm (8.9%; PR: 0.99, 95% CI: 0.79, 1.23) was not significantly lower than those in the control group.

Prespecified adjusted analyses resulted in similar effect estimates of interventions on reported ARI in index children across all measures (Supplemental Table 1). Children in single WASH and combined WSH plus nutrition arms had lower prevalence of reported ARI than those randomly assigned to the combined WSH group (Supplemental Table 2).

In secondary analyses, we observed a similar impact using the more specific outcome, where reported ARI plus fever in index children was lower in the water treatment (3.4%, PR: 0.65, 95% CI: 0.46, 0.93), sanitation (2.8%, PR: 0.54, 95% CI: 0.37, 0.78), handwashing (3.3%, PR: 0.63, 95% CI: 0.44, 0.91), and the combined WSH+N (2.9%, PR: 0.58, 95% CI: 0.40, 0.84) than those in the control arm (5.7%) (Figure 2). However, we observed a reduction in the reported prevalence of panting, wheezing, or difficulty breathing only among index children from the handwashing (198%, PR: 0.58, 95% CI: 0.36, 0.91) and WSH+N arms (2.4%, 95% CI: 0.44, 1.03) compared with the control group (3.5%)

In subgroup analyses, we found that the prevalence of these respiratory illness symptoms was lower in females than males, but there was no significant difference in the PRs across gender (Table 2). We found no differences in the effect of interventions in Year 1 versus Year 2 except in the water and WSH+N arms, where the impact on ARI and ARI plus fever was higher in Year 2 (Supplemental Table 3). The prevalence of ARI varied over the intervention period (Supplemental Figure 1).

**Table 2:** Unadjusted respiratory outcome PRs by child gender, interventions vs. control, among index children in Bangladesh: 1- and 2-year follow-up combined

Males				Females			
Outcome/arm	*N*	Prev	PR (95% CI)	*N*	Prev	PR (95% CI)	Interaction, *P*-value
Cough or difficulty breathing (ARI)							
Control	1,131	9.81	Ref	1,157	7.78	Ref	–
Water	602	7.64	0.76 (0.53, 1.06)	606	4.85	0.64 (0.42, 0.96)	0.45
Sanitation	591	6.94	0.69 (0.48, 0.98)	585	5.81	0.75 (0.51, 1.09)	0.73
Handwashing	578	6.40	0.64 (0.43, 0.96)	584	5.65	0.72 (0.48, 1.09)	0.65
WSH	618	9.22	0.94 (0.68, 1.29)	576	8.51	1.08 (0.78, 1.50)	0.54
Nutrition	593	9.11	0.90 (0.68, 1.18)	566	5.65	0.71 (0.48, 1.07)	0.23
WSH+nutrition	559	6.26	0.63 (0.41, 0.96)	638	5.64	0.72 (0.50, 1.03)	0.61
Panting, wheezing, or difficulty breathing							
Control	1,131	4.07	Ref	1,157	2.94	Ref	–
Water	602	4.15	0.93 (0.55, 1.56)	606	2.31	0.82 (0.43, 1.55)	0.74
Sanitation	591	3.89	0.89 (0.54, 1.50)	585	1.20	0.43 (0.19, 0.97)	0.13
Handwashing	578	2.60	0.56 (0.33, 0.30)	584	1.37	0.53 (0.25, 1.15)	0.92
WSH	618	4.21	0.99 (0.57, 1.17)	576	3.13	1.05 (0.61, 1.81)	0.89
Nutrition	593	3.88	0.86 (0.46, 1.54)	566	1.94	0.71 (0.36, 1.42)	0.63
WSH+nutrition	559	2.86	0.62 (0.32, 1.20)	638	2.04	0.73 (0.39, 1.13)	0.76
Fever and ARI							
Control	1,131	5.75	Ref	1,157	4.41	Ref	–
Water	602	4.32	0.75 (0.46, 1.24)	606	2.48	0.53 (0.30, 0.94)	0.33
Sanitation	591	3.05	0.52 (0.30, 0.90)	585	2.56	0.56 (0.31, 1.00)	0.88
Handwashing	578	3.46	0.60 (0.35, 1.03)	584	3.08	0.67 (0.36, 1.25)	0.77
WSH	618	4.05	0.89 (0.44, 1.08)	576	4.69	1.09 (0.33, 0.74)	0.22
Nutrition	593	5.40	0.92 (0.62, 1.34)	566	3.00	0.65 (0.36, 1.16)	0.29
WSH+nutrition	559	3.58	0.53 (0.30, 0.95)	638	2.35	0.64 (0.41, 0.98)	0.66

ARI = acute respiratory illness; PR = prevalence ratio; WSH = water, sanitation, and handwashing.

## DISCUSSION

In this cluster-randomized trial, reported respiratory illness (ARI) among index children was significantly lower in households that received the sanitation intervention that included regular promotion plus individual latrines, potties, and scoops (28% lower); or chlorinated drinking water intervention (30%), handwashing intervention alone (32%); or all in combination along with nutritional supplements (34%) than those in control households (prevalence: 8.8%). Children randomly assigned to nutrition interventions or combined water, sanitation, and hygiene interventions did not experience fewer respiratory illnesses than children in the control arms.

For handwashing interventions, these findings reinforce well-known protective effects of handwashing on respiratory illness by interrupting pathogen transmission through hands.^5^ Viral infections that are predominantly spread by fomite contact through hands, eyes, or noses are consistently reduced with simple handwashing interventions, as promoted within this trial. This study additionally demonstrates the effectiveness of the handwashing intervention, where homemade soapy water with free detergent refills was promoted with free handwashing stations near the latrine and kitchen.

Prior studies report mixed results on the impact of sanitation and water interventions on respiratory illness.^17,18^ Our study findings add to the literature that demonstrate a reduction in respiratory illness in children from sanitation interventions.^19^ Given that we observed a significant reduction in diarrheal diseases in the sanitation arm in this trial, it is possible that the children potentially benefitted from lower respiratory illness through stronger immune systems and adequate micronutrient levels.^8,13^ We also observed lower fecal indicator bacteria in food and stored water in the improved water arm in this trial, suggesting reduced contamination along direct transmission pathways.^20^ Reduction in ARI from improved water quality requires further investigation into exposure and transmission of relevant waterborne pathogens. Further research into reliable objective biomarkers that can be used in community-based studies could improve pneumonia diagnoses in respondents with cough or nonspecific symptoms.^21^

Malnourished children are at a higher risk of infection including respiratory illness.^2^ The WASH Benefits trial delivered lipid nutrient supplements (LNS) for children between 6 and 24 months while promoting breastfeeding practices and providing micronutrient-rich complementary food. Children in the nutrition intervention groups were taller and had higher weight-for-height Z scores than the children in control households, indicating better nutritional status.^13^ In analyses published elsewhere, compared with the control group, children in the nutrition arms (N and WSHN) had higher prevalence of meeting the minimum dietary diversity score through complementary feeding, which was promoted alongside LNS.^22^ In a subsample at 3 months, 51–55% of women reported exclusive breastfeeding their children in the last 24 hours compared with 18% in the control group.^23^ We did not observe a significant reduction in reported respiratory illness in children from households that received nutrition supplements. When compared with children in control households, those in the single nutrition arm had an 18% lower prevalence of reported respiratory illness (ARI), but the difference was not significant in this trial. A nonsignificant reduction in caregiver-reported respiratory illness morbidity in children following LNS is consistent with results from other studies.^24,25^ We, however, report a significant reduction in reported respiratory illness when nutrient supplements were delivered in households that also received improved water, sanitation, and hygiene interventions. Improving nutritional status of young children may be insufficient to impact respiratory illness in highly contaminated environments.

We have no satisfying explanation for why the combined WSH package did not reduce ARI similar to individual W, S, and H components. One hypothesis could have been that implementation of several interventions together resulted in lower adherence in the combined arm, but this was not corroborated by measures of adherence^16^ or in patterns observed in other infectious disease outcomes.^13^ Notably, we found a significant reduction in reported ARI and fever plus ARI in combined WSH+N households, suggesting implementation of or adherence to a more complex, combined intervention as not a limiting factor here. In any case, our results contribute to findings from other studies that did not detect additive benefits to child health from combining WSH interventions.^26^ Our failure to detect added benefits from combined WASH interventions over single interventions suggests that future studies or programs should consider single targeted interventions to be cost effective.

This study has several limitations. Neither the respondent nor the data collector who surveyed the household conditions was masked to the intervention assignment. Therefore, respiratory illness measured through caregiver-reported symptoms is subject to courtesy bias. The direction of courtesy bias in households that receive interventions is known to inflate health impact when outcome is based on the caregiver-reported prevalence of disease.^27^ Respiratory illness unlike diarrheal disease is less likely to be directly linked to our interventions by the study respondents. Moreover, courtesy bias would not be expected to affect reports in the single water treatment, sanitation, and handwashing promotion arms, but not in the combined arms. We also found no evidence of bias using negative control outcomes in this study, suggesting that differential outcome reporting bias was unlikely.^13^

Second, in the absence of clinical assessments of symptoms, we defined our primary outcome (ARI) broadly as cough or panting, wheezing, or difficulty breathing. This did not allow us to detect changes in more severe respiratory illness such as pneumonia or allow us to compare our estimates with studies that use the WHO definition of pneumonia. We detected somewhat stronger effects in the most specific assessment of ARI plus fever, suggesting that these interventions likely impact severe respiratory illness such as pneumonia (Figure 2). Reported symptoms such as cough, panting, or wheezing or shortness of breath in young children are nonspecific and can indicate noninfectious causes such as asthma. However, these symptoms reflect a burden of illness to a child’s immune system and may increase the risk of pneumonia. The impact on ARI is likely to reflect a genuine interruption of respiratory pathogen transmission because the effect was consistent when assessing more specific respiratory illness (reported fever plus ARI) in the water, improved sanitation, handwashing, and WSH+N interventions compared with the control households.

Water, sanitation, and hygiene interventions that achieved high uptake reduced respiratory illness in young children in rural Bangladesh. The same benefit was observed when water, sanitation, and hygiene interventions were successfully integrated with nutrition interventions. We did not find any additive benefit of combining multiple components of WSH in this study. These findings provide further support for multiple health benefits of water, sanitation, handwashing, and nutrition interventions.

## Supplemental material, tables, and figure

Supplemental materials
