# Supplementary material for: Effect of Improved Water Quality, Sanitation, Hygiene and Nutrition Interventions on Respiratory Illness in Young Children in Rural Bangladesh: A Multi-Arm Cluster-Randomized Controlled Trial
Source: Am J Trop Med Hyg. 2020 Feb 24;102(5):1124–30. doi: 10.4269/ajtmh.19-0769 (PMC7204588; doi:10.4269/ajtmh.19-0769)

Supplemental Material

CONSORT Abstract Checklist

Item	Standard description	Description for Cluster Trials	Multi-Arm Trial Extension	Reported in section
Title	Identification of the study as randomized trial	Identification of the study as cluster-randomized	Identification as a multi-arm randomized trial in the title or an indication of the number of treatment groups that the participants were randomly assigned to	Title
Trial design	Description of the trial design (e.g. parallel, cluster, non-inferiority)	Definition of cluster and description of how the design features apply to the clusters	Specification of the number of treatment groups; details of any groups added or dropped	Methods
Methods				
Participants	Eligibility criteria for participants and the settings where the data were collected	Eligibility criteria for clusters		Methods
Interventions	Interventions intended for each group	Whether interventions pertain to the cluster level, the individual participant level or both		Methods
Objective	Specific objective or hypothesis	Background		
Outcome	Clearly defined primary outcome for this report	Whether outcome measures pertain to the cluster level, the individual participant level or both		Methods
Randomization	How participants were allocated to interventions	Methods		
Blinding (masking)	Whether or not participants, care givers, and those assessing the outcomes were blinded to group assignment	Methods		
Results				
Numbers randomized	Number of participants randomized to each group	Number of clusters randomized to each group		Findings
Recruitment	Trial status	Not applicable – publication indicates		

		study has been completed	
Numbers analyzed	Number of participants analyzed in each group	For each group, number of clusters included in each analysis	Findings
Outcome	For the primary outcome, a result for each group and the estimated effect size and its precision	Results at the individual or cluster level as applicable and a coefficient of intra cluster correlation (ICC or k) for each primary outcome	Findings
Harms	Important adverse events or side effects	Not applicable	Not applicable
Conclusions	General interpretation of the results	Interpretation	Interpretation
Trial registration	Registration number and name of trial register	Methods	Methods
Funding	Source of funding	Funding	Funding

CONSORT Checklist

Section/Topic	Item No.	Standard Checklist item	Extension for cluster designs	Multi-Arm Trial Extension	Reported in section
**Title and abstract**					
	1a	Identification as randomized trial in the title	Identification as cluster randomized trial in the title	Identification as a multi-arm randomized trial in the title or an indication of the number of treatment groups that the participants were randomly assigned to	Title
	1b	Structured summary of trial design, methods, results, and conclusions (for specific guidance see CONSORT for abstracts)[i, ii]	See table 2	Specification of the number of treatment groups; details of any groups added or dropped	Summary
**Introduction**					
**Background and objectives**	2a	Scientific background and explanation of rationale	Rationale for using a cluster design	Rationale for using a multi-arm design	Introduction
	2b	Specific objectives and hypotheses	Whether objectives pertain to the cluster level, the individual participant level or both	Specification of the research question referring to all of the treatment groups	Introduction

				Clear statement of all hypotheses to be tested and the primary comparisons involved	
**Methods**					
**Trial design**	3a	Description of trial design (such as parallel, factorial) including allocation ratio	Definition of cluster and description of how the design features apply to the clusters	Specification of the number of treatment groups	Method [Study design]
	3b	Important changes to methods after trial commencement (such as eligibility criteria), with reasons		Details of any treatment groups added or dropped (if relevant), with reasons, and/or changes to the allocation ratio	Not applicable
**Participants**	4a	Eligibility criteria for participants	Eligibility criteria for clusters		Method [Participants[
	4b	Settings and locations where the data were collected			
**Interventions**	5	The interventions for each group with sufficient details to allow replication, including how and when they were actually administered	Whether interventions pertain to the cluster level, the individual participant level or both		Methods [Procedures]
**Outcomes**	6a	Completely defined pre-specified primary and secondary outcome measures, including how and when they were assessed	Whether outcome measures pertain to the cluster level, the individual participant level or both		Methods [Outcomes]
	6b	Any changes to trial outcomes after the trial commenced, with reasons			Not applicable

**Sample size**	7a	How sample size was determined	Method of calculation, number of clusters(s) (and whether equal or unequal cluster sizes are assumed), cluster size, a coefficient of intracluster correlation (ICC or k), and an indication of its uncertainty	Planned sample size with details of how it was determined for each primary comparison	Methods [Statistical analyses]
	7b	When applicable, explanation of any interim analyses and stopping guidelines			Not applicable
**Randomization**					
**Sequence generation**	8a	Method used to generate the random allocation sequence			Methods [Randomization and masking]
	8b	Type of randomization; details of any restriction (such as blocking and block size)	Details of stratification or matching if used		Methods [Randomization and masking]
**Allocation Concealment mechanism**	9	Mechanism used to implement the random allocation sequence (such as sequentially numbered containers), describing any steps taken to conceal the sequence until interventions were assigned	Specification that allocation was based on clusters rather than individuals and whether allocation concealment (if any) was at the cluster level, the individual participant level or both		Methods [Randomization and masking]
**Implementation**	10	Who generated the random allocation sequence, who enrolled participants, and who assigned participants to interventions	Replace by 10,10b,10c		Methods
	10a		Who generated the random allocation sequence, who enrolled clusters, and who assigned clusters to interventions		Methods [Randomization and masking]

	10b		Mechanism by which individual participants were included in clusters for the purposes of the trial (such as complete enumeration, random sampling)		Methods [Participants]
	10c		From whom consent was sought (representatives of the cluster, or individual cluster members, or both), and whether consent was sought before or after randomisation		Methods [Participants]
**Blinding**	11a	If done, who was blinded after assignment to interventions (for example, participants, care providers, those assessing outcomes) and how			Methods [Randomization and masking]
	11b	If relevant, description of the similarity of interventions			Not applicable
**Statistical methods**	12a	Statistical methods used to compare groups for primary and secondary outcomes		Explicitly state if no adjustments for multiplicity were applied; if adjustments were applied, state the method used	Methods [Statistical analyses]
	12b	Methods for additional analyses, such as subgroup analyses and adjusted analyses			Methods [Statistical analyses]
**Results**					
**Participant flow (a diagram is strongly recommended)**	13a	For each group, the numbers of participants who were randomly assigned, received intended treatment, and were analysed for	For each group, the numbers of clusters that were randomly assigned, received intended treatment, and were analysed for the primary outcome		Results [Fig. 1]

		the primary outcome			
	13b	For each group, losses and exclusions after randomization, together with reasons	For each group, losses and exclusions for both clusters and individual cluster members		Results [Fig. 1]
**Recruitment**	14a	Dates defining the periods of recruitment and follow-up		If periods of recruitment and follow-up are different across treatment groups (eg, groups were added or dropped), the periods of recruitment and follow-up, reason(s) for the differences, and any statistical implications should be described	Results [1st paragraph]
	14b	Why the trial ended or was stopped			Not applicable
**Baseline data**	15	A table showing baseline demographic and clinical characteristics for each group	Baseline characteristics for the individual and cluster levels as applicable for each group		Results [Table 1]
**Numbers analyzed**	16	For each group, number of participants (denominator) included in each analysis and whether the analysis was by original assigned groups	For each group, number of clusters included in each analysis		Results [Fig. 1]
**Outcomes and estimation**	17a	For each primary and secondary outcome, results for each group, and the estimated effect size and its precision (such as 95% confidence interval)	Results at the individual or cluster levelas applicable and a coefficient of intracluster correlation (ICC or k) for each primary outcome	Results for each prespecified comparison of treatment groups	Result [Fig 2, Table 2,3]
	17b	For binary outcomes,			Results [Fig 1]

		presentation of both absolute and relative effect sizes is recommended		
**Ancillary analyses**	18	Results of any other analyses performed, including subgroup analyses and adjusted analyses, distinguishing pre-specified from exploratory		Results [Table 2,3, S1, S2]
**Harms**	19	All important harms or unintended effects in each group (for specific guidance see CONSORT for harms[iii]		Not applicable
**Discussion**				
**Limitations**	20	Trial limitations, addressing sources of potential bias, imprecision, and, if relevant, multiplicity of analyses		Discussion
**Generalizability**	21	Generalizability (external validity, applicability) of the trial findings	Generalizability to clusters and/or individual participants (as relevant)	Discussion, last paragraph
**Interpretation**	22	Interpretation consistent with results, balancing benefits and harms, and considering other relevant evidence		Discussion
**Other information**				
**Registration**	23	Registration number and name of trial registry		Methods [Statistical analyses]
**Protocol**	24	Where the full trial protocol can be accessed, if available		Ref XX
**Funding**	25	Sources of funding and other support, role of funders		Method [Role of funding agency], Acknowledgements

Hopewell S, Clarke M, Moher D, Wager E, Middleton P, Altman DG, et al. CONSORT for reporting randomized trials in journal and conference abstracts. Lancet 2008, 371:281-283
Hopewell S, Clarke M, Moher D, Wager E, Middleton P, Altman DG at al (2008) CONSORT for reporting randomized controlled trials in journal and conference abstracts: explanation and elaboration. PLoS Med 5(1): e20
Ioannidis JP, Evans SJ, Gotzsche PC, O'Neill RT, Altman DG, Schulz K, Moher D. Better reporting of harms in randomized trials: an extension of the CONSORT statement. Ann Intern Med 2004; 141(10):781-788
Juszczak E, Altman DG, Hopewell S, Schulz K. Reporting of Multi-Arm Parallel-Group Randomized Trials: Extension of the CONSORT 2010 StatementCONSORT Extension for Reporting Multi-Arm Parallel Group Randomized Trials CONSORT Extension for Reporting Multi-Arm Parallel Group Randomized Trials. JAMA. 2019;321(16):1610-1620. doi:10.1001/jama.2019.3087

Supplemental Table S1: Adjusted effects of intervention on prevalence of respiratory outcomes, intervention vs control, among index children, Bangladesh: 1 and 2 year follow up combined

Outcome/Arm	N	Prev	PR (95% CI)	P-Value	PD (95% CI)	P-Value
**Cough or difficulty breathing (ARI)**						
Control	2288	8.78	Ref		Ref	
Water	1208	6.29	0.72(.53,0.98)	0.04	-0.02(-0.04,0.00)	0.03
Sanitation	1176	6.38	0.72(0.55,0.94)	0.02	-0.02(-0.04,-0.01)	0.01
Handwashing	1162	6.02	0.70(0.51,0.95)	0.02	-0.03(-0.05,-0.01)	0.02
WSH	1194	8.88	0.99(0.78,1.26)	0.96	-0.00(-0.02,0.02)	0.96
Nutrition	1159	7.42	0.86(0.66,1.12)	0.26	-0.012(-0.03,0.01)	0.25
WSH+Nutrition	1197	5.93	0.67(0.50,0.91)	0.01	-0.03(-0.05,-0.01)	0.004
**Panting, wheezing or difficulty breathing**						
Control	2288	3.50	Ref		Ref	
Water	1208	3.23	0.91(0.62,1.31)	0.59	-0.003(-0.02,0.01)	0.71
Sanitation	1176	2.55	0.74(0.49,1.12)	0.15	-0.01(-0.02,0.003)	0.13
Handwashing	1162	1.98	0.54(0.32,0.91]	0.02	-0.02(-0.03,-0.004)	0.01
WSH	1194	3.69	1.06(0.73,1.53)	0.76	0.002(-0.01,0.02)	0.76
Nutrition	1159	2.93	0.82(0.51,1.22)	0.45	-0.01(-0.02,0.01)	0.43
WSH+Nutrition	1197	2.42	0.67(0.42,1.04)	0.07	-0.01(-0.02,0.00)	0.06
**Fever and ARI**						
Control	2288	5.07	Ref		Ref	
Water	1208	3.39	0.67(0.46,0.93)	0.01	-0.02(-0.03,-0.00)	0.04
Sanitation	1176	2.81	0.53(0.36,0.79)	0.002	-0.02(-0.04,-0.01)	0.00
Handwashing	1162	3.27	0.65(0.41,1.04)	0.07	-0.02(-0.04,-0.00)	0.05
WSH	1194	4.36	0.87(0.63,1.20)	0.40	-0.01(-0.21,0.01)	0.39
Nutrition	1159	4.23	0.84(0.59,1.19)	0.32	-0.01(-0.02,0.01)	0.31
WSH+Nutrition	1197	2.92	0.55(0.39,0.77)	0.001	-0.02(-0.03,-0.01)	0.00

Supplemental Table S2: Respiratory outcome prevalence ratios in index children, Bangladesh comparing combined (intervention) vs. single (reference) arm in combine one and two year follow up

	Unadjusted				Adjusted			
Outcome/arm	Prev. Ratio 95%CI	P value	Prev. Diff 95%CI	P value	Prev. Ratio 95%CI	P value	Prev. Diff 95%CI	P value
**Cough or difficulty breathing (ARI)**								
WSH vs. Water	1.41 (1.06,1.89)	0.02	0.03(0.004,0.05)	0.02	1.38(0.97,1.96)	0.07	0.03(-0.00,0.05)	0.02
WSH vs. Sanitation	1.37(1.04,1.81)	0.03	0.02(0.003,0.05)	0.03	1.38(1.08,1.78)	0.01	0.02(0.00,0.05)	0.03
WSH vs. Handwashing	1.45(1.08,1.94)	0.01	0.03(0.01,0.05)	0.01	1.45(1.03,2.05)	0.03	0.03(0.003,0.05)	0.01
WSHN vs. WSH	0.69(0.51,0.91)	0.01	0.02(0.003,0.05)	0.03	0.68(0.49,0.94)	0.03	-0.028(-0.05,-0.01)	0.01
WSHN vs N	0.83(0.61,1.12)	0.23	0.02(0.01,0.003)	0.03	1.26(0.88,1.79)	0.21	-0.01(-0.01,0.04)	0.23
**Panting, wheezing or difficulty breathing**								
WSH vs. Water	1.15(0.74,1.78)	0.54	0.005(-0.01,0.02)	0.53	1.15(0.73,1.83)	0.56	0.005(-0.01,0.021)	0.55
WSH vs. Sanitation	1.39(0.88,2.18)	0.16	0.01(-0.00,0.02)	0.15	1.41(0.97,2.05)	0.74	0.01(-0.00,0.02)	0.07
WSH vs. Handwashing	1.87(1.13,3.10)	0.01	0.02(0.004,0.03)	0.01	2.02(1.13,3.64)	0.02	0.02(0.004,0.03)	0.01
WSHN vs. WSH	0.69(.43,1.09)	0.11	-0.01(-0.03,0.002)	0.11	0.63(0.41,0.96)	0.03	-0.14(-0.03,-0.00)	0.04
WSHN vs N	0.82 (0.50,1.34)	0.43	-0.005(-0.02,0.01)	0.43	1.25(0.73,2.14)	0.42	0.01(-0.01,0.02)	0.44
**Fev and ARI**								
WSH vs. Water	1.30(0.87,1.96)	0.20	-0.01(-0.03,0.005)	0.20	1.31(0.83,2.05)	0.24	0.01(-0.01,0.03)	0.24
WSH vs. Sanitation	1.56(1.02,2.37)	0.04	-0.02(-0.03,-0.00)	0.04	1.63(.12,2.36)	0.01	0.02(0.00,0.03)	0.04
WSH vs. Handwashing	1.34(0.89,2.02)	0.16	-0.01(-0.03,.004)	0.16	1.35(0.85,2.17)	0.21	0.01(-0.01,0.03)	0.16
WSHN vs. WSH	0.70(0.46,1.05)	0.09	-0.013(-0.03,0.03)	0.09	0.63(0.42,0.94)	0.02	-0.01 (-0.03,0.00)	0.09
WSHN vs N	0.73(0.48,1.10)	0.14	-0.02(-0.03,0.004)	0.14	1.43(0.93,2.21)	0.10	0.01(-0.03,0.00)	0.14

Supplemental Table S3: Unadjusted respiratory outcome prevalence ratios by survey round (year 1, 2), among index children, Bangladesh

	Year 1			Year 2			
Outcome/Arm	N	Prev	PR (95% CI)	N	Prev	PR (95% CI)	Interaction P Value
**Cough or difficult breathing (ARI)**							
Control	1154	8.41	Ref	1134	9.17	Ref	-
Water	614	8.31	0.97(0.67-1.40)	594	4.21	0.46(0.28-0.74)	0.01
Sanitation	586	5.46	0.64(0.43-0.95)	590	7.29	0.79(0.54-1.16)	0.47
Handwashing	591	6.09	0.71(0.47-1.1)	571	5.95	0.65(0.45-0.93)	0.67
WSH	607	9.72	1.14(0.83-1.56)	587	8.01	0.87(0.62-1.22)	0.28
Nutrition	584	7.36	0.85(0.59-1.23)	575	7.48	0.80(0.56-1.15)	0.83
WSH+Nutrition	607	5.58	0.90(0.63-1.27)	590	4.24	0.46(0.29-0.71)	0.01
**Panting, wheezing or difficulty breathing**							
Control	1154	4.42	Ref	1134	2.56	Ref	-
Water	614	4.72	1.03(0.62-1.72)	594	1.68	0.65(0.33-1.29)	0.26
Sanitation	586	2.39	0.54(0.31-0.93)	590	2.71	1.05(0.33-0.56)	0.11
Handwashing	591	2.03	0.47(0.24-0.89)	571	1.93	0.76(0.36-1.57)	0.31
WSH	607	4.28	0.96(0.62-1.49)	587	3.07	1.16(0.66-2.06)	0.59
Nutrition	584	3.08	0.68(0.39-1.19)	575	2.78	1.08(0.55-2.09)	0.20
WSH+Nutrition	607	3.46	0.76(0.44-1.32)	590	1.36	0.51(0.25-1.04)	0.40
**Fever and ARI**							
Control	1154	5.37	Ref	1134	4.76	Ref	-
Water	614	4.89	0.88(0.53-1.46)	594	1.85	0.38(0.18-0.78)	0.06
Sanitation	586	3.24	0.58(0.32-1.04)	590	2.37	0.48(0.26-0.91)	0.69
Handwashing	591	3.21	0.58(0.32-1.04)	571	3.33	0.69(0.41-1.18)	0.61
WSH	607	4.61	0.85(0.55_1.31)	587	4.09	0.86(0.53-1.39)	0.98
Nutrition	584	4.45	0.79(0.49-1.27)	575	4.00	0.81(0.51-1.30)	0.93
WSH+Nutrition	607	3.79	0.71(0.44-1.15)	590	2.03	0.43(0.23-0.80)	0.26

Outcomes were assessed in 4747 index children at the Year 1 follow up (mean age 0.73 years, sd 0.14), and 4667 index children (mean age 1.87, sd 0.17) at the Year 2 follow up

Supplemental Figure S1: 7-day ARI (defined as cough or difficulty breathing) prevalence in index children by calendar month (combined over two-year follow-up period). Individual children were measured only once at each round of follow up; each round took approximately one year. Control and intervention clusters were geographically matched and measured concurrently. The control data series includes on average 191 observations per month (range: 76, 261) and the intervention data series includes on average 591 observations per month (range: 226, 782).

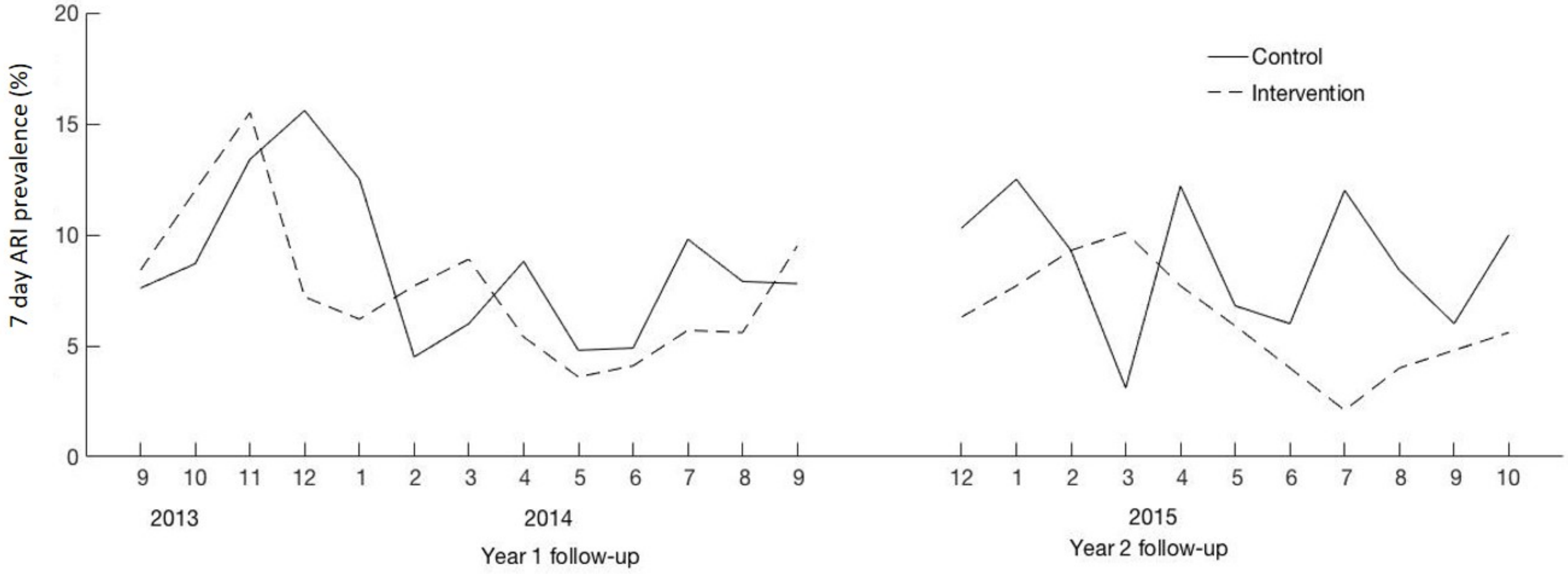

Supplement: Supplementary file 1 [file tpmd190769.SD1.pdf]
